# Roadmap for Advancing Pre-Clinical Science in Traumatic Brain Injury

**DOI:** 10.1089/neu.2021.0094

**Published:** 2021-11-23

**Authors:** Douglas H. Smith, Patrick M. Kochanek, Susanna Rosi, Retsina Meyer, Chantelle Ferland-Beckham, Eric M. Prager, Stephen T. Ahlers, Fiona Crawford

**Affiliations:** ^1^ Center for Brain Injury and Repair, Department of Neurosurgery, University of Pennsylvania, Philadelphia, Pennsylvania, USA.; ^2^ Department of Critical Care Medicine; Safar Center for Resuscitation Research, University of Pittsburgh School of Medicine and Children's Hospital of Pittsburgh of UPMC, Rangos Research Center, Pittsburgh, Pennsylvania, USA.; ^3^ Department of Physical Therapy Rehabilitation Science, Neurological Surgery, Weill Institute for Neuroscience, University of California San Francisco, Zuckerberg San Francisco General Hospital, San Francisco, California, USA.; ^4^ Cohen Veterans Bioscience, New York, New York, USA.; ^5^ Delix Therapeutics, Inc, Boston, Massachusetts, USA.; ^6^ Department of Neurotrauma, Operational and Undersea Medicine Directorate Naval Medical Research Center, Silver Spring, Maryland, USA.; ^7^ The Roskamp Institute, Sarasota, Florida, USA.

**Keywords:** diffuse axonal injury, neurodegeneration, neuroinflammation, neurological dysfunction, pre-clinical animal models, traumatic brain injury

## Abstract

Pre-clinical models of disease have long played important roles in the advancement of new treatments. However, in traumatic brain injury (TBI), despite the availability of numerous model systems, translation from bench to bedside remains elusive. Integrating clinical relevance into pre-clinical model development is a critical step toward advancing therapies for TBI patients across the spectrum of injury severity. Pre-clinical models include *in vivo* and *ex vivo* animal work—both small and large—and *in vitro* modeling. The wide range of pre-clinical models reflect substantial attempts to replicate multiple aspects of TBI sequelae in humans. Although these models reveal multiple putative mechanisms underlying TBI pathophysiology, failures to translate these findings into successful clinical trials call into question the clinical relevance and applicability of the models. Here, we address the promises and pitfalls of pre-clinical models with the goal of evolving frameworks that will advance translational TBI research across models, injury types, and the heterogenous etiology of pathology.

## Introduction

For many decades, traumatic brain injury (TBI) has been recognized as a major global health concern, leading to the development of an almost countless number of pre-clinical TBI models used to characterize mechanisms of TBI and identify therapeutic targets. Although our understanding of TBI patholophysiology has been greatly advanced through these studies, no therapies demonstrating high efficacy in pre-clinical models have shown success in clinical trials.^1^ This report addresses the benefits and weaknesses of pre-clinical TBI models and how their clinical translation can be enhanced.

TBI induces a highly heterogeneous range of pathophysiological responses in humans, posing an enormous challenge for the development of treatment strategies. Indeed, this diverse nature of TBI is thought to have significantly contributed to repeated failures of clinical trials, for which enrollment relied more on symptom severity rather than the underlying causes,^2^ as detailed in a report from a National Institutes of Health (NIH) workshop. Another major target of criticism of the failed clinical trials has been the validity of pre-clinical models to replicate relevant mechanisms of human TBI in the development of therapies. Although some models provide valuable insights into aspects of the pathogenesis of TBI, none have reproduced all, or even most, of the features observed in the corresponding human TBI in either the acute or chronic phases.

Curiously, in certain cases, no comparison to the human condition is attempted or published. Beyond the questions of establishing translational or construct validity, the pre-clinical field is plagued by challenges of reproducibility and robustness in methods and model generation.^3–6^ The inability to develop effective treatment strategies through pre-clinical studies may also be a result of reporting bias (whereby negative results are not published), poor study design, or misinterpretation or over-representation of pre-clinical findings.^7^ Finally, and pragmatically, many human TBI pathologies reflect mechanisms unique to a large gyrencephalic brain, which are not easily replicated in rodents who have relatively small lissencephalic brains.

There is a general consensus that a single model, a singular focus on a subcomponent of the disease, or even a single species cannot recapitulate the broad array of etiologies of symptoms and the functional and pathological sequelae of human TBI. To better understand and treat the human condition, frameworks to assess translational validity and improve the probability of accurately extrapolating pre-clinical data to clinical treatments are needed. With these considerations in mind, and as part of the Brain Trauma Blueprint, TBI State of the Science, we undertook to review the current state of the field with a view to construct a framework that identifies where more research is needed and how researchers and funders alike can make the greatest impact on future TBI treatment translation.

### Consideration of traumatic brain injury biomechanics

The biomechanics of TBI have been extensively characterized in humans, demonstrating wide variation in injury mechanisms and relative contributions of shear, tensile, and compressive strains. However, surprisingly few animal models have undergone a similar characterization. In reality, the production of some models appears driven by the available methodology or desired lesion type rather than using known biomechanical parameters.^8^ Nonetheless, there is a general consensus that parameters should include “dynamic” deformation of the brain by mechanical loading, though how and to what degree that deformation occurs can vary.^9^ The rapid or “dynamic” aspect of the mechanical pulse is important to create a classic viscoelastic response across the brain. To replicate human TBI conditions in pre-clinical models, the mechanical pulse should be ∼≤50 msec.^10^ For example, stretching axons to twice their resting length over hundreds of milliseconds does not cause damage. However, dynamic stretching over even shorter distances causes immediate mechanical damage to the axonal cytoskeleton and physiological dysfunction.^11^

For clinical relevancy, this dynamic mechanical principle has been used across the multi-scale of pre-clinical modeling, spanning computational, *in vitro*, *ex vivo*, and *in vivo* small and large animal models. Designing precise biomechanical modeling (e.g., scaling exposure forces to match those experienced by humans) is especially important, considering that the large size of the human brain has been shown to play an important role in the extent of tissue deformation and damage during dynamic head rotational acceleration. Accordingly, it remains critical to precisely scale these forces for the smaller brains in animal modeling to induce similar pathobiological consequences of human TBI. Although many models have been developed to incorporate relevant biomechanical parameters, there remains a common challenge to measure the extent and distribution of brain tissue deformation.

### Consideration of traumatic brain injury severity

Severity of TBI has wide-ranging consequences on the long-term behavioral and functional outcomes. Although it is possible to mimic human TBI biomechanics in some animal models (e.g., the strain and strain rate needed to induce relevant brain tissue deformation), pre-clinical models are often unable to precisely and accurately model injury severity characterized in human TBI. Further, there is commonly a mismatch between the assessed injury severity in pre-clinical models compared with human TBI. For example, the descriptions of “moderate” or “severe” TBI are often used for rodent models, even though the animals can ambulate, groom, and eat shortly after the injury, which is clearly a very different scenario from typical clinical conditions. In some models, the attribution of severity is often arbitrarily based on the extent of overt brain damage or how close the injury device setting is to a threshold that can induce mortality. Notably, however, mortality in these models typically reflects the extent of damage to the brainstem and not necessarily the extent of injury to the cerebrum.^12,13^

The general use of a singular variable to determine TBI severity, rather than using a multi-modal approach, has come under scrutiny. Siebold and colleeagues reviewed how studies categorized injury severity in mice after controlled cortical impact (CCI) injury.^14^ They identified four main parameters used to define injury severity: 1) injury induction parameters such as depth and impact velocity; 2) tissue loss; 3) motor and cognitive deficits; and 4) injury induction parameters based on previous studies. Interestingly, injury induction parameters, for example, vary widely by severity. Indeed, in looking at the impact depth and velocity of a CCI impactor, the “mild” injuries had a depth of 0.1–1.0 mm and velocity of 3–6 m/s, “moderate” injury had a depth of 0.5–3.0 mm and velocity of 1.5–6 m/s, and “severe” injuries had a depth ranging from 0.5 to 2.0 mm and velocity of 3–6 m/s, indicating a lack of consensus in defining injury severity as a function of depth and velocity across the field. Additional confounders with regard to matching TBI severity in pre-clinical models with human TBI include: 1) differences in brain anatomy, physiology, and neurological outcome measures between animals and humans; 2) the accuracy of the injury device to replicate injury severity; and 3) pre-clinical studies that largely ignore the developmental/aging processes, biological sex, and other comorbidities of human TBI that influence recovery and outcomes.

These considerations highlight the need to develop a consensus for assessing injury severity through multi-modal approaches aimed at attaining a clinical correlate. This may be particularly difficult when modeling mild TBI (mTBI), where no immediate gross damage or pathological changes are induced and the pathobiology evolves over time post-injury. Loss of consciousness and acute responsivity/functionality guide initial determination of TBI severity in humans, but such measures are not easily translated to animal models, particularly when most use anesthesia (see below). Measuring specific, longitudinal time points for recovery, conducting neurological assessments, and exploring structural alterations after injury, reporting procedures to grade the severity of injury, including standardization of surgical parameters (i.e., type of anesthesia used), injury biomechanics, and behavioral and histological end-points, will help to harmonize common data elements of TBI severity across laboratories^15,16^ and reduce opposing or conflicting findings. Further, the goal is to recapitulate in pre-clinical models the *pathobiology* observed in human cases.

Above, we have acknowledged the need to attempt to model biomechanical parameters commensurate with those sustained in human injury, but also the difficulties with this endeavor, given the inherent physiological differences between the human brain and brains of the model systems. Ultimately, across our many model systems, our need is to reproduce pathobiological *consequences* of TBI that can reflect the spectrum of human TBI etiology and severity, enabling deeper interrogation of molecular mechanisms, and provide platforms for therapeutic testing.

The considerations outlined above and the breadth of human TBI heterogeneity suggest that one strategy may be to nominate certain approaches to model particular aspects of human TBI sequelae; we elaborate on these below. Through all this, there needs to be consideration of any host-specific differences that may be relevant to the particular pathogenic mechanism in question (e.g., the apparent need for humanization of proteins relevant to certain proteinopathies is discussed below). However, translational relevance does not end with the model itself; the TBI literature is replete with treatment studies in pre-clinical models that do not adequately address dosing, relevant timing of administration paradigms, bioavailability, pharmacokinetics, or pharmacodynamics for the potential therapeutics under investigation. A discourse on these factors is beyond the scope of this current review, but they warrant critical consideration for therapeutic development in any pre-clinical model.

## Major Pre-Clinical Traumatic Brain Injury Models

The variety of animal models of TBI has historically posed an enormous challenge in comparing data from one laboratory to another, let alone from one species to another. Across the many types of rodent models, the most commonly used techniques include inducing dynamic mechanical deformation of the brain through impact with an impounder or weight on the cortical surface or skull, “fluid percussion” injury (FPI) (pressurized fluid pulse on the cortical surface), and blast exposure. Impact models primarily vary by whether or not the injury is delivered to the skull in a closed head injury, or to the cortical surface through a cranial window, and by having the head either fixed in place or moveable upon impact. Adaptations of these models have also been used to study the effects of repetitive TBI, primarily with the focus on understanding cumulative concussive and subconcussive (where concussive injury is typically used interchangeably with mTBI) blows to the skull or brain. Unfortunately, these devices often have shortcomings that can limit clinical relevance; CCI devices, for example, have been found to make several repeated impacts when the impacter should only hit the skull once, exhibit horizonal movement when in contact with the target, and vary in velocity and depth of impact.^17^ Moreover, the sham control for mice receiving CCI has traditionally been mice receiving craniectomy alone, which in and of itself has been demonstrated to produce outcomes consistent with mTBI.^18^

Other models include rotational acceleration injuries in species with large gyrencephalic brains in order to recapitulate the most common biomechanical aspects of TBI in humans. Primate and swine models have typically used a pneumatic actuator to induce a controlled head rotation that is restricted in one plane, whereas the sheep model uses captive bolt impact on a metal plate, inducing unrestricted head movements. Similarly, TBI from a blast wave in rodents and large animals can be recapitulated in pre-clinical models that are of particular relevance among military personnel because of repeated exposures during military training and in combat. These models have recently been adapted from use primarily in shock tubes to use in open-field spaces to induce low-intensity blast waves that reflect real-world blast propogation parameters.^19^

While not intended to be comprehensive, we provide a few of the major categories of models, how they are created, and their signature sequelae in Table 1. Importantly, lissencephalic and gyrencephalic models each have their benefits and drawbacks when trying to replicate aspects of TBI. To maximize the potential of a successful translation of a pre-clinical therapy into TBI clinical trials, pre-clinical data should be obtained from multiple experiments and in several TBI models, in small and large animals. Indeed, notably, many groups who use large animal TBI models also use rodent models.

**Table 1.** Categories of Major Pre-Clinical TBI Models

Model	Description	Head fixation	Biomechanics	Injury distribution	Signature sequelae	References
*Closed* h*ead* i*mpact* i*njury: rat, mouse (lissencephalic)*	Closed head impact injury avoids craniotomy, but typically delivers a weight drop impact device or impactor (see CCI below) to one side of unprotected skull. Head is placed on a hard surface	Head is typically unconstrained; sometimes a restraint bag is used	Most involve compression of the skull (with possible fracture, depending on force)	Primarily diffuse	Induces diffuse brain injury. However, depending on force of the impactor, induces a range of pathologies, including skull fractures, cerebral edema, BBB dysfunction, axonal injury, neurodegeneration, hemorrhagic lesions, and motor and cognitive dysfunction	^89,132,152–154^
*Fluid* p*ercussion* i*njury (FPI): rat, mouse (lissencephalic); pig (gyrencephalic)*	Midline (central) or lateral injury inflicted by a pendulum striking the piston of a reservoir of fluid to generate pressure pulse to the brain through a craniotomy	Rodent heads are fixed with stereotax; pig's head is constrained with implanted bolts to the FP device with Leuer Lok adaptor	Localized pressure pulse to exposed, intact dura produces brief displacement and mechanical deformation of the brain. Severity of injury is proportional to the force of the pulse	Mixed: Focal and diffuse injuries may result, depending on injury location and severity	Induces mild-to-severe TBI. Causes intracranial hemorrhage, brain swelling, BBB disruption, axonal injury, progressive gray matter damage, inflammation, and motor and cognitive dysfunction Midline (central) FPI: diffuse TBI with bilateral structural injury Lateral FPI: focal contusions (ipsilateral) and hemorrhage and diffuse injury in subcortical and contralateral structures FPI has high mortality compared to other models	^12,155–167^
*Controlled* c*ortical* i*mpact (CCI): rat, mouse (lissencephalic)*	Pneumatic or electromagnetic impact device drives rigid impactor into a surgically exposed brain. Rapid acceleration of rod guided by software that controls the velocity, time, and depth of impact	Fixed with stereotax	Requires preparation of the skull by craniotomy, followed by a strike to the dura mater. Induces mechanical deformation of brain tissue. Mechanical factors (e.g., time, velocity, and depth of impact) is controlled, unlike with FPI that only controls pendulum height	Mainly focal (frontal and temporal regions), but can be diffuse	Focal cortical tissue loss, depending on depth/velocity, hippocampal and thalamic damage, acute subdural hematoma, axonal injury, BBB dysfunction, and motor and cognitive dysfunction Diffuse damage includes hippocampal, brainstem neuronal, and axonal injury	^14,17,119,168–170^
*Closed* head impact model of engineered rotational acceleration (*CHIMERA*)*: rat and mouse, (lissencephalic); ferret (gyrencephalic)*	Delivers high-pressure–driven impact from a metal piston that strikes the dorsal surface of the head. Animals secured in supine position on platform	Body restrained with Velcro straps Head unconstrained	Metal piston strikes dorsal surface of the head, driving the head upward, following a looped trajectory in the sagittal plane. Allows precise control of impact energy, velocity, and direction of injury	Mainly diffuse	Causes axonal injury, neuroinflammation, neurodegeneration, and motor and cognitive dysfunction	^63,65,171–173^
*Penetrating* b*allistic-*l*ike* b*rain* i*njury (PBBI): rat, mouse (lissencephalic)*	Transmission of projectiles with high energy and leading shockwave. Produces temporary cavity in the brain many times larger than the projectile. Variants of model: low-velocity PBBI	Fixed with stereotax	Penetrating injury with force directed perpendicular to injury tract. Causes severe mechanical damage through formation of a visible cavity	Mainly focal	Immediate and subacute changes in intracranial pressure, BBB permeability, and brain edema. Extensive intracerebral hemorrhage and temporary cavity formation, motor and cognitive dysfunction	^174–178^
*Weight-*d*rop Models (e.g., Maryland or Marmarou): rat, mouse (lissencephalic)*	Skull is exposed (with or without craniotomy) to a free falling, guided weight	Body restrained with adhesive tape. Head is unconstrained	Impact on intact skull causes sagittal rotational acceleration (frontal impact [Maryland], Dorsal-ventral [Marmarou]). Injury severity is altered by adjusting mass of the weight and the height from which it falls	Mainly diffuse	Both models induce axonal injury (Marmarou model specifically produces brainstem axonal injury), cerebral edema, and ventriculomegaly. Widespread damage to neurons, axons and microvasculature, and BBB disruption	^36,89,179,180^
*Primary* blast injury*: rat, mouse, (lissencephalic); pig (gyrencephalic)*	Uses compression-driven shock tube or open-field low-intensity blast. Produces non-penetrating supersonic blast-wave loading impulse to simulate mild-to-severe blast effects	Variable: some restrained in a sling, but head unconstrained. Some models constrain the head laterally and inferiorly to prevent acceleration-induced injury	Shock and pressure wave propagation results in biodynamic response, including head acceleration and rotation, body translocation	Mainly diffuse	Sequelae highly dependent upon blast overpressure intensity, which include immediate and subacute changes in intracranial pressure, BBB permeability, and brain edema. Enduring motor, cognitive, and affective effects	^181–185^
*Captive* bolt impact; sheep *(gyrencephalic)*	Uses a captive bolt gun to dynamically impact the head, inducing linear and rotational acceleration	Head unconstrained	Captive bolt impact induces unrestrained head movements with linear and rotational accelerations. High variability in head accelerations and pathologies between animals	A mixture of diffuse and focal injury	DAI, skull fractures, focal contusion, necrosis, and subarachnoid hemorrhage	^186,187^
*Rotational* a*cceleration* m*odel:* p*ig, non-human primate (gyrencephalic)*	Produces non-impact, rapid angular acceleration to induce inertial forces common in human TBI resulting from falls, impact, or collisions	Pig's head secured to rotation acceleration injury apparatus or mechanical rotation device (e.g., HYGE™), depending on model. For the non-human primate, the head is secured with a helmet	Produces purely impulsive non-impact lateral and rotational head movement using different angular planes (coronal, sagittal, and axial) at controlled rotational acceleration levels	Mainly diffuse	In both primates and pigs, dynamic tissue deformation causes DAI as the primary pathology Swine: mild-to-severe TBI, with or without coma or loss of consciousness depending on the level of acceleration and angle of head rotation. DAI, BBB disruption, and hippocampal dysfunction Non-human primates primarily produce severe TBI. Duration of coma associated with the extent of DAI	^24,37,188–191^

TBI, traumatic brain injury; PBBI, penetrating ballistic-like brain injury; FP, fluid percussion; BBB, blood–brain barrier; DAI, diffuse axonal injury.

Within these categories of injury models, researchers have focused on different pathophysiologies of TBI. Some aspects of the condition are recapitulated well, such as neuroinflammation, axonal injury, vasculature dysfunction, brain edema, and acute neuronal death.^20^ In addition, certain clinical symptoms have been successfully modeled, including loss of consciousness, cognitive and affective dysfunction, and motor and sensory dysfunction. However, important clinical outcomes of TBI have proven challenging to model, such as TBI-related neurodegeneration (TReND), of which chronic traumatic encephalopathy (CTE) is one form, behavioral/mood changes, headaches, and sleep disturbances.^21^ A factor that may influence TBI outcomes in pre-clinical models is that the models themselves do not fully recapitulate the trauma conditions in humans. For example, pre-clinical models of mild or post-concussive TBI are often poor predictors of symptoms such as headaches, memory impairment, sleep disruption, concentration issues, etc. In addition, although investigators are better at modeling outcomes in more “severe” injury models, as above, animals are typically ambulatory and grooming shortly after injury, unlike severe TBI in humans where coma or loss of consciousness, as indicated by the Glasgow Coma Scale (GCS) score, represents the core diagnostic criteria (see Brenner and colleagues, this issue).

In addition, ≥85% of the cases of severe TBI in humans include polytrauma,^22^ which may complicate the development of pre-clinical models that usually involve only one component of injury. These collective shortfalls in modeling and interpretation have been blamed, in part, for the failure of clinical drug trials in severe TBI patients, namely advancing therapies shown to be efficacious in these limited rodent models after equally limited characterization of drug behavior in the models. Here, we explore the state of science, with a focus specifically on the aspects of TBI that have been successfully modeled pre-clinically, given that these models will assist in understanding how to better bridge pre-clinical research toward clinical treatments.

## Pre-Clinical Modeling of Traumatic Brain Injury Sequelae

### Diffuse axonal injury/traumatic axonal injury

Swollen axonal profiles spread in a multi-focal pattern across the white matter is characteristic of diffuse axonal injury (DAI). This is a diagnostic term in human TBI and also used for studies involving models with gyrencephalic brains,^23,24^ which is more reflective of the distribution of axonal pathology. In contrast, the term traumatic axonal injury (TAI) is used in models with lissencephalic brains because of their relatively sparse white matter^25–27^ and for *in vitro* studies that examine injured axons. In recent years, there has been increased interest in this pathology because of its role as one of the most important pathological features of mTBI or concussion.^28^ However, there have also been increased misunderstanding and disagreements regarding modeling DAI, especially with regard to its biomechanical origins.

For >60 years, DAI has been noted to be a prominent feature of all severities of human TBI.^29^ However, because of the typical lack of mortality after concussion, there has been only one subacute human neuropathological study of an isolated concussion, where DAI was identified as the only pathological change.^28^ Nonetheless, non-invasive techniques are beginning to be used to identify DAI in humans with parallel corroboration studies in pre-clinical models. In particular, advanced neuroimaging has identified similar connectivity changes in white matter tracts in both human concussion and certain pre-clinical models.^30–32^ In addition, emerging blood biomarker analyses have identified axonal proteins and protein fragments that reflect axonal degeneration in humans and animal models.^33,34^ Notably, the animal models allow for the confirmation of axonal pathology in the brain as the likely source for these changes, providing a rationale for diagnosing DAI in humans.^32,35,36^

As noted above for many forms of neuropathological changes in animal modeling, the size of the brain is an important factor, which is particularly important in the development of DAI. It has long been established that mass effects during head rotational acceleration induce shear and tensile forces in the brain tissue, causing selective injury to white matter axons. It is thought that the high organization of axons in white matter tracts, and their fine and very elongated morphology, renders them particularly vulnerable to disruption under these dynamic mechanical forces. Accordingly, researchers have developed head rotational acceleration TBI models using non-human primates,^24^ pigs,^20,37^ and sheep,^38^ which all have relatively large gyrencephalic brains with extensive and anisotropic white matter tracts. This line of research began with seminal studies in non-human primates, where Gennarelli and colleages demonstrated that the “inertial” TBI model replicates the key pathological features of DAI observed in humans and was linked to the induction of immediate coma.^20,24,39^ The models have more recently demonstrated that the angle of head rotation and distribution of axonal pathology is key to the production of immediate coma or transient loss of consciousness, providing a better understanding of clinical outcomes in humans.

Although clinical relevance has been established in large animal models of head rotational acceleration, thus far, these models are not suited for comprehensive studies necessitating large groups. Therefore, potentially more expedient rodent models have been developed, albeit with some debate. Although rodent head rotational acceleration models have been shown to induce selective axonal pathology, missing from the model descriptions is how the Holbourn scaling relationship was addressed.^40^ This calculation has been used across large animal species to determine how to scale the rotational forces to induce the same tissue deformations as occur in human TBI. For example, the forces must be increased 500% for the 140-g brain of a baboon and 600% for a 90-g pig brain to induce similar DAI as found in human TBI, which has been confirmed by histopathological examinations.^41^ By extension, the scaled inertial forces necessary to produce equivalent tissue strains in the ∼2-g brain of a rat would be unachievable at 8000%. However, the mismatch between low rotational forces and histopathological findings has rarely been addressed. In addition, other factors may have contributed to the observed TAI in rodent models. For example, mechanical coupling of the rotation device with the head or impact pressure of weights or impounders could rapidly distort the skull during injury, thereby serving as the primary cause of tissue deformation and axonal pathology as opposed to rotational acceleration. However, this possible alternative mechanical mechanism of injury to the rodent brain is rarely discussed.

Regardless, the debate of the parameters of head rotation necessary to induce TAI does not preclude the use of rodent models to study TAI in TBI as long as the caveats of translation to specific human injuries are kept in mind. Indeed, decades ago, Povlishock and colleagues transformed our understanding of acute TAI using rodent impact models of TBI.^42–44^ The model was also shown to be useful to evaluate therapies for TAI, and many of the evolving axonal pathologies were found to have clinical relevance. Since then, evolving axonal pathology has been identified across many rodent TBI models and can be induced with or without fixation/rotation of the head. Multiple rodent studies have demonstrated progressive axonal changes that have been shown to have broad clinical implications,^45–47^ such as identifying the activation of proteases that degrade the axonal cytoskeleton,^48^ thereby revealing a therapeutic target.^2,34^ More recently, multiple mTBI models have emerged that induce axonal injury in the absence of other overt pathologies, providing another platform for potential translation. For example, without apparent head acceleration/rotation or bodily movement, exposure to low-intensity primary blast waves may disrupt axonal transport and cause myelin sheath defects.^49^

Complementing large and small animal models, *in vitro* models of TAI or computational models allow for controlled biomechanical input parameters,^50–54^ and the evolution of the pathophysiology can be viewed in real time or simulated based on experimental data and/or human findings. These models have shown a spectrum of pathological changes, including immediate breaking of axonal ultrastructure and loss of ionic homeostasis. Specifically, rupture of axonal microtubules causes transport interruption and accumulation of transported cargoes in varicose swellings similar in appearance to the swollen axonal profiles in human DAI. In addition, *in vitro* trauma induces immediate and massive influx of sodium and calcium ions that respectively disrupt electrical signaling and activate proteases, causing secondary damage to the axonal ultrastructure.^39^ Models include classic dynamic stretch injury to micropatterned axonal tracts and dissociated neuronal networks as well as slice and organotypic cultures that are specifically designed to produce axonal injury without neuronal death.^55^ Neurons in culture could serve as a powerful means for high-throughput analyses that are not possible in animal models. Such studies would complement more in-depth analyses in small and large animal models.

Considering that selective axonal injury is one of the most common pathologies of TBI, this unique pathology provides an attractive targert for treatment. Indeed, the NIH held a workshop to examine potential therapies for DAI based largely on evidence from pre-clinical models^56^ in order to facilitate potential translation through targeted clinical trials. Notably, recent advances of blood biomarker analyses of TBI patients in parallel with pre-clinical studies appear to represent a non-invasive tool to diagnose degenerative DAI. Indeed, identification of a high concentrations of axonal proteins and protein fragments in the blood after injury may provide a surrogate marker of axonal degeneration in the brain.^30^ Refinement of these diagnostic measures through parallel studies in human TBI and relevant animal TBI models could help the development of a logical patient enrollment strategy for therapies targeting DAI.

### Neuroinflammation

Neuroinflammation is a prominent feature in the acute and chronic effects of TBI in humans and in pre-clinical models of TBI.^57^ This process includes the activation of brain resident cells, such as microglia and astrocytes, and the recruitment of peripheral immune cells (neutrophils, macrophages, and T cells) in response to the release of inflammatory mediators within the brain. Most of the TBI models currently used are generally thought to recapitulate many aspects of human neuroinflammation after TBI. Indeed, multiple markers of neuroinflammation in human TBI are found across animal models of TBI, including components of both innate and adaptive immunity. Given that these changes appear to be associated with TBI outcomes and are present in human TBI, these models can provide a clinically relevant platform to delineate mechanisms of TBI-related neuroinflammation and provide an opportunity to explore therapeutics that may ameliorate or engage these pathways.

In addition to a primary inflammatory response within the central nervous system (CNS), TBI can induce systemic peripheral immune activation and suppression. Because of the compromised blood–brain barrier (BBB) found in both human TBI and pre-clinical models,^39,58^ infiltrating inflammatory cells and cytokines can access the CNS and aggravate the pathogenesis of TBI. Compelling research has now revealed the significant role of the peripheral immune system and how it interacts with CNS inflammation, which was not a focus in early pre-clinical TBI studies. Thus, the contribution of peripheral inflammation to many of the observations from traditional experimental paradigms (e.g., CCI) may have been underestimated. Indeed, peripherally derived monocytes (C-C motif chemokine receptor 2 [CCR2]⁺) propagate to the injured brain in response to C-C motif chemokine ligand 2 signaling and exacerbate the cognitive impairment in chronic TBI.^59,60^ Such monocyte infiltration accompanying the cognitive deficit after TBI becomes amplified in aged animals.^61^ Moreover, it has been reported that this peripheral trafficking of proinflammatory monocytes in TBI can be treated with a CCR2 antagonist.^59^ Altogether, these studies implicate the peripheral immune system as a key target for probing TBI pathology and its treatment.

Accordingly, ample consideration should be given to the different aspects and timing of neuroinflammatory mechanisms that are likely contributing to both neurodegenerative and -reparative processes after TBI. To this end, accumulating evidence has provided a better understanding of the full spectrum of inflammation post-injury (including myeloid cells, lymphocytes, the microbiome, the vagal response, and spleen),^62–67^ and pre-clinical efforts with direct clinical applications have advanced these complex interactions between peripheral and central immune systems in the pathology and treatment of TBI.

Neuroinflammation persists in the brains of patients for years and even decades after moderate-to-severe TBI and is found in the presence of ongoing neurodegenerative changes, including progressive axon degeneration, white matter atrophy, tauopathies, and amyloid-beta (Aβ) pathologies.^39^ Although prolonged neuroinflammation (>3 weeks after injury) has been observed in multiple pre-clinical models, including, but not limited to, non-penetrating blast injury,^68^ closed head injuries,^62,65,69^ and repetitive injuries,^25,70^ future pre-clinical work will be needed to continue to explore the chronic evolution of neuroinflammation and its potential roles in mitigating and/or promoting progressive neuropathological changes. This may provide therapeutic targets of specific neuroinflammatory responses across broad therapeutic windows of opportunity. For clinical translation, development of new tools and identification of appropriate patient populations (see Pugh and colleagues,^71^ this issue) will all be needed in order to effectively translate research findings in TBI-related neuroinflammation to the clinic. In sum, a number of studies have supported the use of pre-clinical animal models to define and target the chronic inflammatory consequences of TBI from single or multiple injuries, and this area merits considerable additional exploration.

### Vascular injury

Thus far, it appears that pre-clinical animal models can recapitulate the vascular consequences of all severities of human TBI with considerable reliability.^72^ Cerebral vascular injury after TBI can vary in magnitude and scope depending on the severity of injury, in association with many other pathological changes.^73^ Injury to the vasculature can range from BBB dysfunction in the absence of hemorrhage to loss of autoregulation of cerebral blood flow (CBF) to vascular disruption in the form of microhemorrhages^74,75^ to macrohemorrhages and thrombi formation. BBB disruption is common after TBI in both humans and across pre-clinical models—supporting the fidelity of this pathobiology, although it is increasingly recognized that compromised BBB integrity reflects a single component of potential subtle changes in the complex neurovascular unit. Investigations of the vascular consequences of mTBI (or repetitive mTBI) have received increased attention over the past 5–10 years.^76^ These studies are important given that some of the refractory consequences of these milder versions of brain injury are linked, in part, to vascular dysfunction.^77^ In a chronic repetitive mTBI model in mice, involving two mTBIs per week for 3 months, a study demonstrated a significant reduction in CBF at 3 months after last injury.^78^ Using the same injury paradigm, differences in compromised cerebrovascular reactivity were demonstrated at 3 and 9 months after last injury,^79^ reflecting findings in a human moderate-to-severe TBI patient population.^80^

BBB disruption has also recently been shown after mTBI in swine, with the same appearance, albeit to a much lesser extent, as found in moderate-to-severe TBI in humans, although the rete mirabile in swine may limit applicability to human TBI.^58,73^ Finally, disturbances in vascular regulation after TBI can lead to uncoupling of CBF and metabolism and make the brain highly vulnerable to secondary insults, such as hypotension, potentially establishing the brain state that exacerbates or enables long-term pathologies. These findings are also modeled with considerable fidelity across species and TBI models.^81^ The relatively recent emphasis on the role that disruptions of the glymphatic system may play adds to the complexity of changes in the vascular and perivascular space that may contribute to understanding TBI sequelae and provide targets for therapy.^82^

### Metabolic disturbances

Changes in cellular and tissue metabolism are expected across all forms of injury. However, TBI may pose a particularly unique case, attributable to the extremely high blood flow through brain tissue (20% total cardiac output) and predisposition of the brain to oxidative and excitotoxic insults. Seminal studies focused on the metabolic consequences of severe TBI identified a marked suppression in oxidative metabolism that was associated temporally with the presence of coma.^83^ Subsequently, a pre-clinical study outlined the acute and subacute neurometabolic cascade after moderate-to-severe TBI that included acute neurotransmitter release and ionic flux (potassium and calcium), producing a state of hyperglycolysis linked to N-methyl-D-aspartate receptor activation.^84^ Although ischemia can be observed early in severe TBI, it is not required for this hyperglycolysis response. In rodent TBI models, the acute hyperglycolysis is transient, lasting 1–2 h, and is followed by a state of metabolic depression, which can be protracted. There is fidelity for this observation from rodent models to humans, although the time course for these events appears to be more protracted in human TBI. Even in the absence of coma, early studies using fluorodeoxy glucose positron emission tomography scanning revealed an acute increase in glycolysis followed by delayed metabolic suppression in 5 patients with GCS scores in the moderate or mild range, which included vascular injuries identified on computed tomography.^85^ Therefore, these, and other metabolic signatures, may represent important therapeutic targets in TBI, as recently reviewed.^86^

Regarding pre-clinical modeling, the choice of anesthetic importantly influences brain metabolism and can cloud studies of TBI.^87^ Indeed, anesthesia alone has been shown to be neuroprotective in most models.^88^ This has led to the development of models where TBI is induced without anesthesia. However, approval by ethics committes is obviously challenging for these models. In addition, it remains unknown whether the absence of anesthesia more closely mimics the clinical environment.^89^

In delayed or chronic phases after TBI, including mTBI, less work has focused on cerebral metabolic dysregulation; however, studies have reported loss of coupling between CBF and metabolism as assessed by functional magnetic resonance imaging, which is associated with persistent neurological symptoms such as working memory deficits in patients.^90^ Recent studies in a mouse model using closed-TBI concussive impact injury have shown an association between microvascular injury, tauopathy, and behavioral deficits.^91^ A variety of therapeutic strategies might be able to mitigate these metabolic derangements—the approach that has received the greatest level of investigation includes strategies using alternative fuels, such as ketones or lactate,^92^ or therapies targeting mitochondrial dysfunction.^93^

### Acute neuronal death and chronic atrophy

In humans, severe TBI typically involves extensive neuron death in the acute setting, whereas in a neuropathology study of mTBI where DAI was identified, cell death was absent.^28^ Similar findings have been reported in animal models of TBI, with the extent of neuron death, or its absence, also related to injury severity.^20^ Through unknown mechanisms, the acute events of TBI can also trigger progressive neuron and glia cell death and axonal degeneration, leading to expanding loss of tissue volume in humans and rodent models. However, there are substantial differences in the appearance of this progressive atrophy. Only a subset of human TBI cases have been shown to have progressive atrophy, and this typically appears as generalized tissue loss, which is accompanied by ventriculomegaly.^46,94–96^ In contrast, in rodent fluid percussion or CCI causing cortical contusions, progressive tissue atrophy is consistently found. These expanding lesions are typically found to extend outward from a contusion site, with the site of tissue loss filled by an expanding syrinx that eventually joins with the ventrical system.^97^ Accordingly, it remains unclear whether the mechanisms of progressive cell loss in human and rodent TBI are the same. Potentially, anatomical differences of gyrencephalic versus lissencephalic brains may partially account for the different patterns of progressive atrophy after TBI.

Many mechanisms of cell death have been elucidated in animal models of TBI, which appear to have clinical relevance.^98^ Acute neuronal death can result from a myriad of mechanisms in TBI ranging from direct cellular disruption, energy failure, excitotoxicity, various neuronal death programmatic pathways, inflammation, and loss of connectivity.^20^ Studies in multiple rodent models of TBI have revealed significant contributions of delayed neuronal death pathways, such as apoptosis and other programmed cell death pathways,^99^ which appear to correspond to findings in human TBI.^100^ Over the past decade, studies have shown that other neuronal death pathways are involved after TBI in pre-clinical models and represent therapeutic targets; these pathways include necroptosis,^101^ pyroptosis,^102^ ferroptosis,^103^ and autophagy,^104^ among others. The quantitative contribution of each of these pathways likely depends on the injury type, severity, and other factors. Finally, studies have shown that some neurons exhibit characteristics of multiple cell-death pathways. Taken together, the link between acute cellular death in the brain after TBI and chronic neurodegeneration remains to be fully explored, particularly given that some of these mechanisms (such as autophagy) are important to both acute and chronic neurodegeneration and repair.

### Neurodegeneration

Clinically, TBI has been entwined with increased and accelerated neurodegeneration and can induce forms of TReND, most notably, CTE.^21,105^ However, this area has been more difficult to model in the pre-clinical space. Some studies have shown that TBI may be associated with excess amounts of the proteins Aβ and phosphorylated tau (p-tau), which are also implicated in many neurodegenerative diseases.^21,105^ Preliminary evidence has demonstrated progressive and widespread tau pathology in mouse models of severe TBI^106^; however, tau pathology is not a simple phenotype, and the presence or absence of tau phosphorylation alone does not fully define tau pathology and may represent a transient effect of TBI. For example, accumulation of p-tau in axons after TBI is a marker of transport interruption, but not a hallmark tau pathology described for neurodegenerative diseases. Moreover, TBI-dependent amyloid or tau pathologies have been less reliably demonstrated in models of mild or repetitive mTBI, despite the use of mice expressing human amyloid precursor protein or tau proteins,^107^ and any persistence or progression of these pathologies after injury(s) has rarely been demonstrated.^78,106^ This is in striking contrast to human TReND, which is typically identified years after injury.^21^ Moreover, animal models also do not reflect associated features of tau pathology, such as transactive response DNA binding protein 43 (TDP-43) immunoreactive nuclear inclusions. Ongoing and future work will need to consider using transgenic mice with either human tau, amyloid precursor protein, or TDP-43 to potentially facilitate recapitulation of human phenotypes.^108–110^ Clearly, other factors contribute to neurodegeneration in pre-clinical models, given that chronic cognitive deficits have been reported to persist after TBI in the absence of neuronal death or overt tau, amyloid, or other proteinopathy pathology.^70,111,112^

It is important to note that the distribution of certain TReND pathologies in humans, such as pathognomonic CTE tau pathologies observed at the depths of sulci, cannot be reproduced in lissencephalic rodent brains. Beyond rodents, researchers have observed Aβ and tau pathologies in swine TBI with no genetic manipulation.^58,113–115^ In addition, other pathways may be related to the observed neurodegeneration in humans with TBI, and thus further efforts are needed to fully explore the mechanisms related to the chronic effects of TBI on neurodegeneration.^116^ The pathologies responsible for cognitive dysfunction in rodent models of mTBI, where overt neuronal death does not occur, remain to be fully understood, and a deeper exploration of neuronal function/dysfunction is needed. In addition to neuronal death, it may be important to examine associated synaptic function, plasticity, and rewiring in TBI.

### Cognitive dysfunction

Cognitive dysfunction represents a debilitating consequence of TBI that severely impacts quality of life and long-term survival. Irrespective of the severity of injury (e.g., low, intermediate, and high blast levels), degrees of behavioral and cognitive dysfunction (including motor, learning, memory, and emotion- and anxiety-like behaviors) become apparent within 7 days in many pre-clinical models.^117^ Because the injury leading to TBI so often comes from impacts on the front of the head, it primarily affects functions related to the pre-frontal and temporal lobes, including higher cognitive functions such as processing speed, problem solving, decision making, cognitive flexibility, sociability, risk-taking behavior, and working memory functions. Only a few studies using rodent models have measured these higher cognitive functions chronically after injury. After FPI in rats and CCI in mice that led to hippocampal damage, there was associated memory dysfunction as demonstrated in both the Morris water maze and radial arm water maze.^59,62,118,119^ Working memory and mental flexibility measured with a modified Barnes maze was reported to be impaired in mice after closed TBI.^59,62^ Other approaches included using a pre-frontal contusion model in mice, with one study demonstrating recapitulation of persistent cognitive inflexibility using a rule-shift assay typical of human TBI measured with the Wisconsin Card Sorting Test.^120^

Another group demonstrated persistence of spatial memory dysfunction in the Barnes maze over 24 months after a five-hit repetitive mTBI paradigm administered at 3 months of age; this group also showed behavior associated with disinhibition or risk taking in the elevated plus maze.^25^ Although several studies assess a single behavioral outcome, it is important to note that symptom presentation in humans is heterogeneous and complex; in reviewing the literature, Song and colleagues noted that behavioral and emotion-like behavioral changes in rodents could also be observed acutely and chronically and could provide important clues and insight into the outcomes and recovery.^117^ Thus, a battery of cognitive outcome tests (such as the Glasgow Outcome Scale-Extended, Cognitive Failures Questionnaire, or Rivermead Post-Concussion Symptom Questionnaires used in human studies) might better reflect clinical functional recovery, although many other functional outcome domains are now being assessed in human TBI across the injury spectrum and should be back-translated into pre-clinical measures.

### Anxiety-like behaviors

Importantly, long-term consequences of human TBI can include neuropsychological sequelae, such as post-traumatic stress disorder (PTSD), anxiety disorders, depressive disorders, and other “mood” disruptions that cannot be readily modeled pre-clinically. Focusing on a specific symptom or endophenotype of neuropsychological sequelae may be the best approach to addressing some of these consequences. Some TBI studies have tested neuropsychological sequelae of TBI, such as anxiety-, sociability-, impulsivity-, and depression-like behaviors, using traditionally utilized behavioral tests such as the forced swim test, tail suspension test, three-chamber sociability test, and elevated plus maze. For example, using a repetitive mild injury model, characterized by full rotational acceleration of the head, recent studies recapitulated many of the higher cognitive deficits observed in humans, such as poor social behavior, increased risk-taking behavior, and poor working memory.^63,65^ Other studies have reported the development of PTSD-like behaviors months after repetitive blast mTBI injury.^121,122^ To ultimately develop therapeutics that can address these sequelae, these tests should be extended to, and validated in rodent models by demonstrating relevance with human TBI outcomes.

### Sleep

Sleep patterns of many TBI victims are affected regardless of injury severity or age and can lead to insomnia,^123^ awakenings, daytime fatigue, and sleep disordered breathing (commonly described as sleep-wake disturbances [SWDs]).^124–127^ SWDs of any type are reported in between 30% and 70% of all TBI survivors, are one of the most common complaints of mTBI patients, and are associated with impaired functional outcomes, decreased participation in activities, reduced quality of life, and impaired recovery.^128–130^ Rodent models of TBI produce disturbances in sleep and wakefulness that are similar to those in human patients,^128^ though research on SWDs in rodents remains in its infancy. However, from the published research thus far, it is generally agreed upon that SWDs in rodent models manifest as insomnia, excessive daytime sleepiness, and pleiosomnia. However, to date, no well-defined models of sleep-related breathing disorders, circadian rhythm disorders, or abnormal movements during sleep have been developed.^128^ Currently, weight drop is the primary injury model to assess SWDs in rodents,^131–133^ though CCI- and FPI-induced TBI also lead to disturbances such as less wakefulness during the dark phase, increased sleep fragmentation, and spectral changes in theta/alpha ratios, non-REM sleep time, and delta power.^134–143^ Although the majority of the findings across studies are consistent (see Sandsmark and colleagues,^128^ for a review), Sandsmark and colleagues astutely note that methodological differences in the type and severity of injury, heterogeneity of animal response, species/strain, animal sex and age, the time points in which sleep is examined (e.g., acute, subacute, or longitudinal studies), and other methodological factors contribute to the observed inconsistencies.

Sleep is an important translational construct to study because it represents a directly modifiable therapeutic target that shows promise for improving patients' neurological outcomes as well as their overall quality of life. Indeed, one study attempted to translate bedside to bench back to bedside and improve methodological considerations. Modarres and colleagues assessed individual slow waves during sleep and wake states using quantitative electroencephalography (QEEG) channels of patients and mice (exposed to FPI) with mTBI. Although both species showed persistent sleep disturbances, including an inability to maintain wakefulness and more slow waves during wakefulness, demonstrating a strong translational approach, this study had a number of outcomes present in humans that could not be recapitulated in mice (e.g., theta/beta ratios). One potential reason for differences in the findings could be that mice were given 7 days to recover from their injury before QEEG assessments, whereas the human subjects were, on average, 58 months out from their injuries at the time of the experiments and were in an inpatient rehabilitation program.^137^ The investigators also did not appear to take into account comorbid symptoms in humans, such as substance abuse or other neuropsychiatric disorders. Despite shortcomings, including limitations the investigators addressed, sleep disturbances after TBI in pre-clinical models continue to be a promising translational marker, but additional work, including understanding the local changes in neuronal activity and the mechanisms underlying slow wave changes, is needed.

## Additional Considerations of Pre-Clinical Modeling and Clinical Relevance

Just as some forms of TBI are not well understood clinically, there is a paucity of pre-clinical models of certain endophenotypes or tests to create these phenotypes, especially some of the individual variables and outward manifestations of TBI observed in chronic patient populations. One inherent drawback to all animal models of TBI is that they are designed to produce a relatively homogenous injury type, and thus one animal model is unlikely to capture the complex clinical heterogeneity observed in humans, even of one injury type such as a motor vehicle accident or blast exposure. The majority of pre-clinical models are conducted in rodents; these have been successfully used to investigate specific aspects of human TBI (i.e., biomechanical, cellular, and molecular). However, most current studies use animals that are healthy, of a single sex, the same age, and under identical housing and feeding conditions and do not follow the neuropathological or -behavioral trajectory past the acute injury stage. Indeed, experiments often start with all male individuals from inbred lines with little experiential or genetic variability. Thus, without incorporating the complex state of the individual before the TBI and the heterogeneity of the TBIs themselves, many efforts fail in their translatability. A few labs have addressed sex as a biological variable,^63,144,145^ modified select genotypes (e.g., apolipoprotein E [APOE]),^146^ evaluated age differences (both age at injury and age at evaluation post-injury),^112^ and examined effects of either single prolonged stress or unpredictable stress exposures in relation to TBI outcomes.^147,148^

In addition to modeling and measuring likely comorbid conditions before TBI, effective pre-clinical models need to assess the altered functionality caused by the condition in question. Research using larger animal models, which have more clinically relevant human-like brains, face additional challenges. For instance, high-fidelity behavioral methods similar to those in rodents do not exist, limiting the application of these models to the neurobehavioral aspects of the human condition. Additionally, though a broad range of sophisticated neuropsychological tests are available for use in non-human primates, these models face additional ethical challenges that make their use in TBI research unlikely. Nevertheless, translational capability may increase with better pre-clinical models or the refinement of existing pre-clinical models and the use of robust, validated, and translationally relevant behavioral tests. Importantly, non-behavioral cross-domain end-points, such as neuroimaging and blood biomarker analyses, could translate more readily and be run in parallel with human studies.

## Frameworks to Bridging the Translational Divide: Looking Forward

To overcome barriers in pre-clinical studies and improve validity of the studies, we must start with standardization of the models. By establishing standards for the design and conduct of research, experimental biases and other factors will be mitigated, thereby promoting robust, reproducible, valid, and translatable animal-based research outcomes. This process has already begun with programs such as Operation Brain Trauma Therapy (OBTT), which has generated guidelines for designing and enhancing pre-clinical TBI consortia and serves as a potential template for testing multiple therapies across multiple models with multiple outcomes. Through a multi-center approach, OBTT is screening potential therapies in different rodent models as well as in micropigs across a variety of end-points, including behavior, cognitive testing, and histology.^149^ A protocolized, consortium-based approach can also greatly strengthen scientific rigor, which has been an important concern in pre-clinical research.

Similarly, the Moody Project for Translational TBI Research has developed recommendations on optimal approaches to consortium design for pre-clinical testing. In a symposium held in 2016, experts and stakeholders identified limitations and gaps in current pre-clinical TBI studies to improve the translation of promising therapies. The recommendations lay a framework for study design. The framework begins by first measuring pharmacokinetics/pharmacodynamics and brain penetrance of a therapy and selecting a dose, route, and time of administration. Efficacy screening using these parameters would ultimately follow in order to advance precesion diagnostic and individualized therapies with optimization in multiple models.^150^

Finally, and most critically, the National Institute of Neurological Disorders and Stroke (NINDS) has also taken steps to improve pre-clinical study design and data harmonization for TBI and thus maximize the chance of translational success. The NINDS has recently developed pre-clinical TBI Common Data Elements (CDEs) to: standardize and harmonize data collection and analysis from pre-clinical models across centers and bring forth stability with enough flexibility to update as new discoveries are made and reduce variability in how the diverse range of TBI models are evaluated.^16^ In addition, new CDEs on outcome evaluations have recently been released. These guidelines will facilitate reporting, data sharing, comparison of results, and collaboration.^15^ These efforts all build on national and international efforts to improve reproducibility and robustness of pre-clinical TBI research across neuroscience fields.^3,151^

It is anticipated that pre-clinical TBI studies will continue to span numerous models and approaches. However, the landscape will be changing with these new initiatives to encourage broad adoption of standardized approaches and emerging guidelines for translational research. As designed, this harmonization will increasingly provide context to compare results between laboratories and determine their potential clinical relevance. For clinical relevance, we summarize our findings with three takeaways and, as part of our roadmap, four trackable recommendations for next steps that include specific action items (Table 2).

**Table 2.** Actionable Research Recommendations for Pre-Clinical Studies in TBI

Recommendation	Action
*Comprehensive assessment of the validity of accepted animal models*	• Conduct comprehensive assessments within existing models across the core neurophysiological and -psychiatric sequelae of TBI.
	• Consider how population characteristics affect the etiology of the course and outcome of TBI.
	• Support the performance of cross-species validation of phenotypes/models.
	• Integrate appropriate animal models of mTBI and repetitive mTBI for assessment of new rehabilitation approaches across laboratories, particularly with incorporation of long-term outcome assessments.
*Cross-validate and standardize behavioral tests in animal models to recapitulate deficits found in humans after TBI*	• Develop models that account for relevant patient variables, such as previous history of TBI, age, sex, and comorbidities (e.g., PTSD, substance abuse, and major depressive disorder).
	• Develop validated and standardized behavioral tests that can reliably recapitulate higher cognitive deficits displayed by humans, especially in larger non-rodent models such as ferrets and porcine.
	• Consider genetic manipulation to model particular aspects of human TBI, such as humanized tau or TDP-43 to reliably recapitulate TBI-dependent proteinopathy or APOE genotype to incorporate the risk of poor recovery post-TBI.
*Evaluate candidate therapies to assess acute and chronic effects of TBI across multiple models*	• Screen putative therapies on a variety of relevant models in order to assess interventions based on different endophenotypes.
	• Incorporate assessments of brain pharmacokinetics and pharmacodynamics along with confirmation of target engagement when testing therapies—this should be pursued in both forward and reverse translation.
	• Evaluate therapies in pre-clinical models using strategies that expand upon the traditional early post-TBI administration—including assessment of acute and/or chronic administration.
*Standardize experimental variables to improve research rigor, transparency*, *and reproducibility and guide future model development*	• Adopt new pre-clinical Common Data Elements to improve standardization of data collection.
	• Apply best practices for reproducibility and quality assurance in model generation.
	• Ensure that negative data are published or available and searchable in a database.
	• Promote data sharing of pre-clinical data under FAIR principles.

TBI, traumatic brain injury; mTBI, mild TBI; PTSD, post-traumatic stress disorder; TDP-43, transactive response DNA binding protein 43; APOE, apolipoprotein E; FAIR, Findability, Accessibility, Interoperability, and Reusability.

## Conclusion

Major takeaways:

- No single pre-clinical model of TBI will translate to all human TBI pathobiology. Data should be interpreted with this limitation in mind, and clinical trials looking to advance translational findings should be designed to include the patients with the most relevant etiologies to those models as well as have patient etiologies back-translated to animal models.
- Potential therapies should be assessed in multiple relevant models of TBI (i.e., different injury paradigms and species) before advancing to human clinical trials where the heterogeneity of etiology will be a major confounder. The design of such pre-clinical therapeutic studies should also consider clinical translation in terms of timing of treatment administration.
- The predictive validity, and therefore clinical relevance, of animal models of TBI pathologies needs to be confirmed through better communication (e.g., models are developed against clinical and neuropathological observations in the patient population) between clinicans and scientists. Again, this is not expected to be a comprehensive or exhaustive translation, but a well-characterized construct that is consistently and robustly found across the translational divide.
